# Prevalence and persistent use of psychotropic drugs in older adults receiving domiciliary care at baseline

**DOI:** 10.1186/s12877-019-1126-y

**Published:** 2019-04-25

**Authors:** Marie Turmo Lornstad, Marte Aarøen, Sverre Bergh, Jūratė Šaltytė Benth, Anne-Sofie Helvik

**Affiliations:** 1 Department of Public Health and Nursing, Norwegian University of Science and Technology (NTNU), Postbox 8905, N-7491 Trondheim, Norway; 2 Centre for Old Age Psychiatric Research, Innlandet Hospital Trust, Ottestad, Norway; Norwegian National Advisory Unit on Ageing and Health, Vestfold Hospital Trust, Tønsberg, Norway; 3 Institute of Clinical Medicine, University of Oslo, Norway; Health Services Research Unit, Akershus University Hospital, Lørenskog, Norway; Centre for Old Age Psychiatric Research, Innlandet Hospital Trust, Ottestad, Norway; 4 General Practice Research Unit, Department of Public Health and Nursing, Faculty of Medicine and Health Sciences, Norwegian University of Science and Technology (NTNU), Trondheim, Norway; St Olavs University Hospital, Trondheim, Norway; Norwegian National Advisory Unit on Ageing and Health, Vestfold Hospital Trust, Tønsberg, Norway

**Keywords:** Cognitive impairment, Elderly, Home care, Medication, Long term care, Transferral

## Abstract

**Background:**

Little is known about the use of psychotropic drugs in older adults receiving domiciliary care. The first aim was to describe the prevalence and persistency of use of psychotropic drugs in older adults (≥ 70 years) with and without dementia receiving domiciliary care. Furthermore, the second aim was to explore factors associated with persistent drug use at two consecutive time-points. Lastly, we aimed to examine if use of psychotropic drugs changed after admission to a nursing home.

**Methods:**

In total, 1001 community-dwelling older adults receiving domiciliary care at inclusion participated in the study. Information about psychotropic drug use was collected at baseline, after 18 months and after 36 months. The participants’ cognitive function, neuropsychiatric symptoms (NPS) and physical health were assessed at the same assessments. Participants were evaluated for dementia based on all gathered information. Formal level of care (domiciliary care or in a nursing home) was registered at the follow-up assessments.

**Results:**

Prevalence and persistent use of psychotropic drugs in older adults receiving domiciliary care was high. Participants with dementia more often used antipsychotics and antidepressants than participants without dementia. The majority of the participants using antipsychotic drugs used traditional antipsychotics. Younger age was associated with higher odds for persistent use of antipsychotics and antidepressants, and lower odds for persistent use of sedatives. Severity of NPS was associated with persistent use of antidepressants. The odds for use of antipsychotics and antidepressants were higher in those admitted to a nursing home as compared to the community-dwelling participants at the last follow-up.

**Conclusion:**

There was a high prevalence and persistency of use of psychotropic drugs. The prevalence of use of traditional antipsychotics was surprisingly high, which is alarming. Monitoring the effect and adverse effects of psychotropic drugs is an important part of the treatment, and discontinuation should be considered when possible due to the odds for severe adverse effects of such drugs in people with dementia.

**Electronic supplementary material:**

The online version of this article (10.1186/s12877-019-1126-y) contains supplementary material, which is available to authorized users.

## Background

Prevalence of psychotropic drug use is high in older adults (> 70 years) [1, 2]. It is known that the prescription rate of psychotropic drugs in western countries increases with higher age [3, 4], and community-dwelling older adults are prescribed approximately half of all psychotropic drugs prescribed in Norway [4]. A review of 67 studies from the USA concluded that one in four older adults used psychotropic drugs with an abuse or dependency potential (e.g. benzodiazepines) [3]. The prevalence of use of anxiolytics and sedatives increase with age in older adults [5].

In community-dwelling older adults, people with dementia more often use psychotropic drugs than those without dementia [6, 7]. Neuropsychiatric symptoms (NPS) following dementia are very common in community-dwelling older adults receiving domiciliary care and in nursing home residents [8, 9]. Chan et al. reported that in a group of community-dwelling older adults, a clinical diagnosis of dementia, but not the severity of NPS, was associated with the use of psychotropic drugs [10]. Nursing home residents with dementia more often use antipsychotics than residents without dementia [11–13], and the severity of their NPS is associated with persistent use of psychotropic drugs [14]. However, non-pharmacological interventions should be the first-choice treatment of NPS in dementia [15–18], but with severe NPS psychotropic drug use may be unavoidable.

Antipsychotics are commonly used to treat agitation in older adults with dementia, for example a recent study reported the prevalence of antipsychotic use to be 38% [18]. There is little evidence that long term use of antipsychotics is effective in the management of NPS in older adults with dementia, but it may have adverse effects [19], e.g. higher risk of cerebrovascular events [20, 21], increased risk of falling [22, 23] and a higher risk of mortality [24, 25]. Atypical antipsychotics are known to have less extrapyramidal side-effects than traditional antipsychotics [26], but they are also associated with a higher risk of hip fractures and a higher risk of mortality in patients with dementia [27, 28]. Antidepressants and benzodiazepines may also cause serious short- and long-term adverse effects, e.g. increase in falls [22, 29–31]. While prevalence and persistency of psychotropic drugs in nursing home residents have been studied and found to be high [9, 14], it remains to be studied whether persistent use of psychotropic drugs in older adults receiving domiciliary care is related to having dementia and NPS.

Studies have found that the strongest indicators for nursing home admission are functional impairment, cognitive impairment and older age [32–35]. It is known that older adults in nursing homes frequently are prescribed psychotropic drugs [9, 14, 36]. There might be changes in prescription of drugs after admission to nursing home, especially when it comes to psychotropic drugs [36]. One British longitudinal study has linked information about psychotropic drug use in older adults to transition to a nursing home, and found psychotropic drugs to be prescribed more often after admission to a nursing home than among those who continue to live at home [37], but the study did not adjust for dementia and other health conditions. To our knowledge, there are few studies on prescription of psychotropic drugs following admission to long-term nursing home care [36, 37].

Thus, the first aim of the present study was to describe the prevalence of psychotropic drug use in persons ≥ 70 years with or without dementia receiving in-home domiciliary care: at the baseline of the study, after 18 months and after 36 months. The second aim was to assess the persistency in use of psychotropic drugs, and explore factors possibly associated with such persistency, i.e. severity of dementia, degree of NPS, physical health and/or nursing home-admission. The third aim was to explore whether admission to a nursing home, was associated with use of these drugs adjusting for severity of dementia, NPS and physical health.

## Methods

### Study design

This is a longitudinal study with a 36-month follow-up period. The baseline data was collected between August 2008 and December 2010. Follow-up assessments were conducted 18 months and 36 months after baseline-assessment.

### Participants

1796 persons (age ≥ 70 years) from 19 municipalities in the eastern part of Norway were invited to participate in the study. We chose participants over the age of 70, as it in Norway, and internationally, has been discussed changing the limit for being an “old adult” from 65 years or older, to 70 years or older [38]. The participants had to be community-dwelling and receiving domiciliary care from the municipality at inclusion. The amount and kind of service received were not important. Those eligible for inclusion had to have a next of kin who saw them at least once every week. Both established recipients and new recipients of domiciliary care were included. New recipients were recruited successively. The participants were recruited from municipalities of various sizes, both rural and urban.

Of the total 1796 invited persons, 795 declined to participate in the study. Thus, 1001 persons were included. Those who declined were more often women and older than those who participated in the study [8].

### Measures

*The use of drugs* was registered from the participants’ medical records. Drugs were divided into groups according to the Anatomical Therapeutic Chemical (ATC) Classification System. Psychotropic drugs were categorized into; antipsychotics (N05A except lithium), anxiolytics (N05B), hypnotics/sedatives (N05C), antidepressants (N06A), and anti-dementia drugs (N06D). Use of drugs was dichotomized into yes or no. There were no participants with missing information about use of drugs. Dosage of drugs was not available in the present study.

*Physical morbidity* was evaluated using the General Medical Health Rating Scale (GMHR), considering the patients number and severity of medical conditions and the use of drugs due to these conditions. GMHR is scored from 1 to 4, where 1 indicates very poor physical health and 4 indicates good physical health [39].

*The personal activities of daily living (P-ADL)* were measured by the Physical Self-Maintenance Scale (PSMS), evaluating six different basal needs. Each PSMS item is scored from 1 to 5, where higher scores indicate lower P-ADL function [40].

*Cognitive function and dementia severity symptoms* were evaluated by the following tools: Mini-Mental State Examination (MMSE), Clock-Drawing Test (CDT), the Informant Questionnaire on Cognitive Decline in Elderly (IQ-CODE) and Clinical Dementia Rating scale (CDR). MMSE is a standardized test of cognitive function that is scored from 0 to 30 points. Higher scores indicate good cognitive function [41]. The Clock Drawing Test is scored from 0 to 5. A perfect clock is scored 5 [42]. In the Informant Questionnaire on Cognitive Decline in Elderly (IQ-CODE), the next of kin assesses the change in cognitive function during the last decade. The scoring scale goes from 0 to 5. A score < 3 indicates improvement, > 3 indicates decline and a score of 3 indicates no change in cognitive function [43]. The CDR includes six domains; memory, orientation, judgment and problem solving, community affairs, home functions, and personal care. An algorithm gives a total score of 0, 0.5, 1, 2 or 3, indicating respectively; no dementia, possible dementia, and mild, moderate and severe dementia [44]. In the present study, we used CDR sum of boxes (CDR-SoB), with a point score ranging from 0 to 18, where a higher score indicates higher severity of dementia [45]. The assessment tools for cognitive function and severity of dementia have been translated and validated in Norwegian [46–48].

*Neuropsychiatric symptoms (NPS)* were evaluated using the 10-item Neuropsychiatric Inventory (NPI) [49] in a translated Norwegian version [50]. The following 10 symptoms are covered: delusion, hallucination, euphoria, agitation/aggression, disinhibition, irritability/lability, depression/dysphoria, anxiety, apathy/indifference, and aberrant motor behaviour. Each symptom was rated by the next of kin based on its occurrence the previous four weeks.

*Dementia* was diagnosed independently by two experienced physicians in geriatric psychiatry at all three assessments based on all available information. Dementia was diagnosed according to the ICD-10 criteria [51]. In cases of disagreement, consensus was reached after consulting a third clinical expert.

*The formal level of care* at the follow-up assessments was registered as location, i.e. community-dwelling receiving domiciliary care or living in a nursing home. Type of home support at baseline was registered as nursing care, domestic help and/or other types of support such as food delivery, day care centre and safety alarm. Demographic data including age, gender, municipality of residence and marital status were registered in the baseline assessment.

### Procedure

The process of collecting data material was led by a research nurse that cooperated with the assessors in the different municipalities. The majority of the assessors were nurses, social educators and occupational therapists. All the assessors had a two-day course of training on how to use the assessments scales before the baseline data collection. A one-day training course was conducted prior to the second and third assessment. The participants and their next of kin were interviewed simultaneously by two separate assessors.

### Data analysis

Sample characteristics at baseline were presented as means and standard deviations or as frequencies and percentages. Participants with dementia and participants without dementia were compared. Prevalence and persistence of medical drugs by dementia/no dementia and admission to nursing home/living at home were presented as percentages. Due to participants belonging to municipality, data might exhibit hierarchical structure. Therefore, groups of participants were compared by linear mixed model (for continuous data) or generalized linear mixed model (for categorical data) with fixed effects for dementia status and random intercepts for municipality, if necessary.

The use of each defined category of psychotropic drugs given use one or two time-points earlier (Lag 1 and Lag 2, respectively) was assessed by a generalized linear mixed model with random intercepts for municipality. The dependent variable was use of the given psychotropic drugs at A2 or A3, while the independent variable was use of the same category of psychotropic drugs at baseline. Each model was adjusted for pre-defined covariates measured at baseline (age, sex, marital status, CDR, GMHR, PSMS, affective NPS, psychosis NPS and agitation NPS) and then reduced for excessive covariates by applying Akaike’s Information Criteria (AIC) [52], where a lower value indicates a better model.

To assess factors associated with persistent use of each category of psychotropic drugs, a generalized linear mixed model with random effects for patients nested within municipality was estimated. Dependent variable was defined as persistent use if the given category of psychotropic drugs was used at two consecutive time-points. Adjustment for covariates measured at a previous time-point was performed. Relative importance of each covariate in the adjusted models was determined from standardized regression coefficients. In addition, effect of location on use of psychotropic drugs at the same time-point was assessed by estimating the model with random effects for patients nested within municipality and fixed effects for location and interaction between time and location. The results were adjusted for covariates measured at baseline (age, gender) or at the same time-point (health measures and marital status). The results in the adjusted models were presented as odds ratio (OR) and 95% confidence intervals (CI).

The data was analysed in SPSS version 24 and SAS version 9.4. All tests were two-sided. Results with *p*-values below 0.05 were considered statistically significant.

## Results

### Sample characteristics

**Fig. 1:**
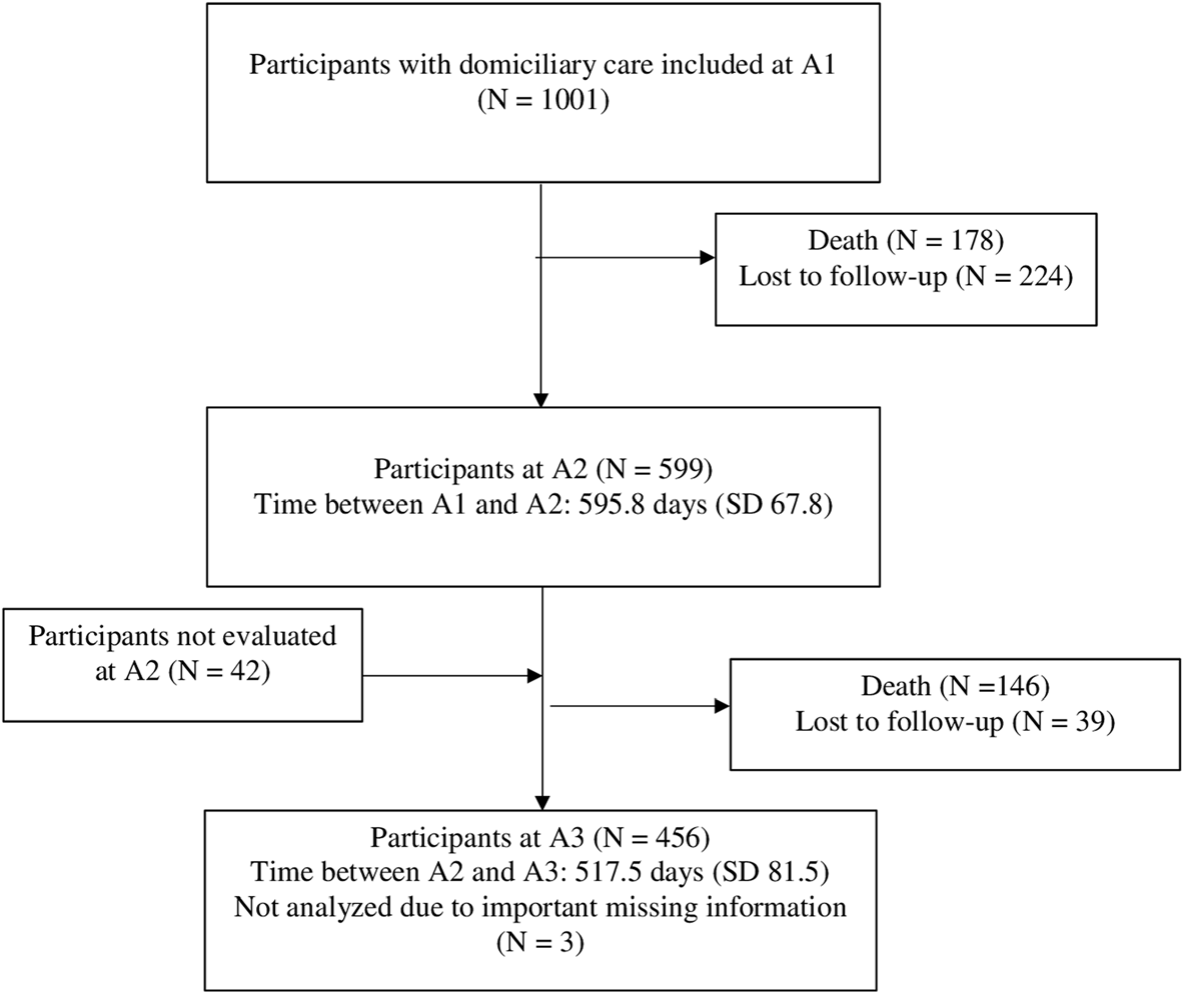
Flow chart of participants from baseline (A1) to last follow-up (A3) with a mean (SD) follow-up time at each assessment

As shown in Table 1, the mean (SD) age of the total sample of 1001 participants at baseline was 83.4 (5.7) years, 683 (68.2%) were female and 703 (70.2%) were single or a widow/widower. Of the participants, 599 (59.8%) were assessed at the second assessment (A2), and 456 (45.5%) were available for the third assessment (A3) (Fig. 1). At A2 and at A3, 89 (14.9%) and 114 (25.2%) had been admitted to a nursing home, respectively.

**Table 1:** Sample characteristics at baseline

		Total (*N* = 1001)	Dementia (*N* = 415)	No dementia (*N* = 586)	p-value
*Socio-demographics*					
Age	Mean (SD)	83.4 (5.7)	84.5 (5.6)	82.6 (5.6)	**< 0.001** ^a^
Females	N (%)	683 (68.2)	273 (65.8)	410 (70.0)	0.171^b^
Married	N (%)	297 (29.7)	131 (31.6)	166 (28.4)	0.250^b^
*Health condition*					
GMHR^d^					
Good	N (%)	155 (15.5)	43 (10.4)	112 (19.1)	**< 0.001** ^b^
Fairly Good	N (%)	392 (39.2)	147 (35.5)	245 (41.9)	
Poor	N (%)	346 (34.6)	166 (40.1)	180 (30.8)	
Very Poor	N (%)	106 (10.6)	58 (14.0)	48 (8.2)	
PSMS^e^	Mean (SD)	9.2 (3.5)	10.9 (4.0)	7.9 (2.6)	**< 0.001** ^a^
NPI Agitation sub-syndrome^f^	Mean (SD)	1.6 (4.4)	2.6 (5.7)	0.9 (2.9)	**< 0.001** ^a,c^
NPI Psychosis sub-syndrome^g^	Mean (SD)	0.5 (2.0)	1.1 (2.8)	0.2 (1.1)	**< 0.001** ^a,c^
NPI Affective sub-syndrome^h^	Mean (SD)	2.9 (5.3)	4.8 (6.8)	1.6 (3.3)	**< 0.001** ^a^
No of drugs	Mean (SD)	5.3 (2.9)	5.4 (3.0)	5.3 (2.9)	0.699^a^
Type of domiciliary care^i^					
Nursing care	N (%)	670 (67.4)	347 (84.0)	323 (55.6)	**< 0.001** ^b^
Domestic help	N (%)	528 (53.1)	210 (50.8)	318 (54.7)	0.229
Other types of support	N (%)	599 (59.8)	272 (65.5)	327 (55.8)	**0.001** ^b^

Agitation sub-syndrome: Agitation/aggression, euphoria, disinhibition, irritability/lability, aberrant motor behavior.

Psychosis sub-syndrome: Delusions, hallucinations

Affective sub-syndrome: Depression/dysphoria, anxiety, apathy/indifference

*GMHR* General Medical Health Rating, *PSMS* Physical Self-Maintenance Scale, *NPI* Neuropsychiatric Inventory

^a^Linear mixed model

^b^Generalized linear mixed model

^c^No cluster effect on municipality level

^d^Missing information in 2 participants

^e^Missing information in 5 participants

^f^Missing information in 35 participants

^g^Missing information in 26 participants

^h^Missing information in 29 participants

^i^Missing information in 7 participants

### Prevalence of psychotropic drugs by dementia and place of living

The prevalence of psychotropic drug use is presented in Table 2. Sedatives were the most frequently used drugs at all time-points (varying between 21 and 23%). Antipsychotics were the least frequently used types of psychotropic drug, being 4% at the first (A1) and 7% at the last time-point (A3). The majority of the participants using antipsychotics used traditional antipsychotics, and traditional antipsychotics were also more frequently used by participants with dementia than by participants without dementia at all time-points. The prevalence of use of antidepressants was approximately 16% at A1, and 22% at A3. The prevalence of use of any psychotropic drug was high in all three assessments. Antipsychotics, antidepressants and anti-dementia drugs were more frequently used in those with dementia than in those without dementia at all time-points. Furthermore, we found the prevalence of use of all types of psychotropic drugs, except from sedatives at A2, to be higher in those admitted to a nursing home than in those living at home at A2 and A3 (Table 3).

**Table 2:** Prevalence and persistence in use of psychotropic drugs

	Prevalence n (%)					
	A1		A2		A3	
	All	D/nD	All	D/nD	All	D/nD
	(N = 1001)	(N = 415/586)	(*N* = 599)	(*N* = 304/295)	(*N* = 453)	(*N* = 227/226)
Psychotropic drug use						
Antipsychotics (AP)	36 (3.6)	24/12 (5.8/2.0)**	28 (4.7)	22/6 (7.2/2.0)**	30 (6.6)	26/4 (11.5/1.8)***
Traditional AP	31 (3.1)	19/12 (4.6/2.0)*	23 (3.8)	17/6 (5.6/2.0)*	23 (5.1)	19/4 (8.4/1.8)**
Atypical AP	5 (0.5)	5/0 (1.2/0)	6 (1.0)	6/0 (2.0/0)	8 (1.8)	8/0 (3.5/0)
Antidepressants	155 (15.5)	82/73 (19.8/12.5)**	121 (20.2)	80/41 (26.3/13.9)***	101 (22.3)	70/31 (30.8/13.7)***
Anxiolytics	86 (8.6)	43/43 (10.4/7.3)	71 (11.9)	48/23 (15.8/7.8)**	53 (11.7)	35/18 (15.4/8.0)*
Sedatives	221 (22.1)	103/118 (24.8/20.1)	128 (21.4)	78/50 (25.7/16.9)*	103 (22.7)	59/44 (26.0/19.5)
Anti-dementia drugs	57 (5.7)	53/4 (12.8/0.7)***	60 (10.0)	57/3 (18.8/1.0)***	34 (7.5)	31/3 (13.7/1.3)***
Any PTD	403 (40.3)	208/195 (50.1/33.3)***	272 (45.4)	178/94 (58.6/31.9)***	210 (46.4)	132/78 (58.1/34.5)***
	Persistence n (%)					
	A1-A2			A2-A3		
	All	D/nD		All	D/nD	
Psychotropic drug use						
Antipsychotics (AP)	17 (63.0)^a^	12/5 (63.2/62.5)		13 (65.0)^a^	10/3 (62.5/75.0)	
Traditional AP	13 (54.2)	8/5 (50.0/62.5)		11 (64.7)	8/3 (61.5/75.0)	
Atypical AP	3 (100)	3/0 (100/0)		1 (25.0)	1/0 (25.0/0)	
Antidepressants	83 (82.2)	45/38 (83.3/80.9)		69 (82.1)	41/28 (80.4/84.8)	
Anxiolytics	29 (63.0)	17/12 (70.8/54.5)		22 (57.9)	14/8 (53.8/66.7)	
Sedatives	77 (66.4)	37/40 (68.5/64.5)		52 (67.5)	27/25 (71.1/64.1)	
Anti-dementia drugs	26 (74.3)	24/2 (75.0/66.7)		22 (66.7)	20/2 (64.5/100)	
Any PTD	191 (81.3)	106/85 (86.9/75.2)*		142 (83.5)	85/57 (85.9/80.3)	

*A1*: Assessment 1, at baseline

*A2:* Assessment 2, 18 months after baseline

*A3*: Assessment 3, 36 months after baseline

*D* Dementia, *nD* No dementia, *PTD* Psychotropic drugs

* *p* < 0.05; ** *p* < 0.01; *** *p* < 0.001; *p*-values were calculated by generalized linear mixed model adjusting for municipality level if present

^a^One participant changed between traditional AP and atypical AP between assessments, which is why the numbers of persistent users of traditional AP plus persistent users of atypical AP does not equal persistent users of antipsychotics. This is the case for A1-A2 and A2-A3

**Table 3:** Prevalence in use of psychotropic drugs according to nursing home admission

	A2	A3
	NHA/no NHA	NHA/no NHA
	(*N* = 89/510)	(*N* = 114/339)
Psychotropic drug use		
Antipsychotics (AP)	11.2 / 3.5**	18.4 / 2.7***
Traditional AP	7.9 / 3.1*	12.3 / 2.7***
Atypical AP	4.5 / 0.4**	7.0 / 0
Antidepressants	33.7 / 17.8**	43.0 / 15.3***
Anxiolytics	20.2 / 10.4*	21.1 / 8.6**
Sedatives	25.8 / 20.6	32.5 / 19.5**
Anti-dementia drugs	23.6 / 7.6***	20.2 / 3.2***
Any PTD	67.4 / 41.6***	73.7 / 37.2***

*A2:* Assessment 2, 18 months after baseline

*A3*: Assessment 3, 36 months after baseline

*NHA* Nursing home admission, *no NHA* Living at home, *PTD* Psychotropic drugs

* p < 0.05; ** p < 0.01; *** p < 0.001; *p*-values were calculated by generalized linear mixed model adjusting for municipality level if present

### Persistent use of psychotropic drugs by dementia

The persistent use of psychotropic drugs was high for all types of psychotropic drugs from A1 to A2 and from A2 to A3, both in participants with and without dementia (Table 2). Persistent use of any psychotropic drug was over 80% for the total sample, and it was higher between A1 and A2 in participants with dementia than in participants without dementia.

Odds for use of psychotropic drugs at one time-point, given use of the same type of psychotropic drug at earlier time-points, are presented in Table 4. In general, the odds for persistent use were highest when compared with the nearest time-point (Lag 1), but lower when compared to two time-points earlier (Lag 2). The adjusted OR for use of antipsychotics decreased the most, from 80.5 (28.9; 224.0) to 20.8 (5.8; 75.0), but the odds for the other drugs were also markedly reduced.

**Table 4:** OR for use of each category of psychotropic drugs at one time-point given use one or two time-points earlier, respectively, lag 1 and lag 2, covariates were measured at baseline

Variable	Number of users		Unadjusted		Adjusted^b^	
	Baseline	A2	OR (95% CI)	p-value	OR (95% CI)	p-value^o^
Lag 1 (*N*=565^a^)						
Antipsychotics^c^	26	27	76.8 (28.5; 207.2)	< 0.001	80.5 (28.9; 224.0)	< 0.001
Antidepressants^d^	97	118	53.3 (28.6; 90.1)	< 0.001	47.3 (25.1; 89.1)	< 0.001^p^
Anxiolytics^e^	44	67	25.9 (12.0; 55.5)	< 0.001	23.6 (10.7; 52.1)	< 0.001
Sedatives^f^	109	118	17.3 (10.5; 28.6)	< 0.001	18.2 (10.9; 30.4)	< 0.001
Lag 2 (N=432^a^)						
	Baseline	A3				
Antipsychotics^i^	17	29	24.2 (7.1; 83.1)	< 0.001	20.8 (5.8; 75.0)	< 0.001
Antidepressants^j^	66	97	29.8 (15.0; 59.2)	< 0.001	28.1 (13.8; 57.4)	< 0.001^p^
Anxiolytics^k^	32	50	10.4 (4.5; 23.9)	< 0.001	11.4 (4.5; 28.7)	< 0.001
Sedatives^l^	74	98	6.6 (3.8; 11.3)	< 0.001	6.6 (3.8; 11.4)	< 0.001

*Lag 1*: Two consecutive assessment time points (baseline and A2)

*Lag 2*: One time point between selected time points (baseline and A3)

^a^ Cases with at least one missing on covariates were excluded

^b^Following adjustment variables considered: Age, gender, marital status, CDR-SoB, GMHR, PSMS, NPI Affective sub-syndrome, NPI Psychosis sub-syndrome, NPI Agitation sub-syndrome all measured at A1 (baseline); Nursing home admission was not included as adjustment variable (no baseline values available); both models reduced by AIC

^c^Adjusted for Gender

^d^Adjusted for CDR-SoB and NPI Affective sub-syndrome

^e^Adjusted for PSMS and NPI Affective sub-syndrome

^f^Adjusted for NPI Affective sub-syndrome and NPI Agitation sub-syndrome

^g^Adjusted for PSMS

^h^Adjusted for CDR-SoB and NPI Psychosis sub-syndrome

^i^Adjusted for Gender and PSMS

^j^Adjusted for CDR-SoB, NPI Affective sub-syndrome and NPI Psychosis sub-syndrome

^k^Adjusted for Marital status, GMHR and NPI Affective sub-syndrome

^l^Adjusted for Age and CDR-SoB

^m^Adjusted for Marital status and CDR-SoB

^n^Adjusted for Gender, Age, Marital status and CDR-SoB

^o^*p*-values were calculated by generalized linear mixed model adjusting for municipality level if present

^p^ no cluster effect at municipality level present

### Factors associated with persistent use of psychotropic drugs at two consecutive time-points

In the adjusted analysis, an increased severity of affective sub-syndrome of NPI, lower P-ADL function (higher PSMS), admission to nursing home and younger age were associated with higher odds for persistent use of antidepressants (Table 5). Furthermore, younger age was associated with higher odds for persistent use of antipsychotics, and lower odds for persistent use of sedatives. Female gender and lower P-ADL function were associated with higher odds for persistent use of anxiolytics.

**Table 5:** OR for use of each category of psychotropic drugs at one time-point given use of the same drug at the previous time point with covariates measured at the same previous time-point

Variable	Antipsychotics (*N* = 954)				Antidepressants (N = 954)			
	Unadjusted		Adjusted		Unadjusted		Adjusted	
	OR (95% CI)	*p*-value	OR (95% CI)	p-value	OR (95% CI)	p-value	OR (95% CI)	p-value
Assessed at previous time-point								
CDR-SoB	1.14 (1.04; 1.24)	**0.005**	1.04 (0.91; 1.19)	0.582^4^	1.13 (1.07; 1.19)	**< 0.001**	1.04 (0.96; 1.13)	0.346^5^
GMHR (Good/fairly good)	0.56 (0.26; 1.22)	0.145	1.08 (0.43; 2.73)	0.866^9^	0.91 (0.59; 1.40)	0.668	1.37 (0.84; 2.23)	0.214^6^
PSMS	1.17 (1.07; 1.28)	**0.001**	1.13 (0.99; 1.29)	0.057^2^	1.13 (1.07; 1.20)	**< 0.001**	1.10 (1.01; 1.19)	**0.025** ^3^
NPI Agitation sub-syndrome	1.07 (1.00; 1.14)	**0.049**	1.03 (0.94; 1.13)	0.557^5^	1.05 (1.00; 1.10)	**0.037**	0.97 (0.91; 1.02)	0.248^8^
NPI Psychosis sub-syndrome	1.10 (0.97; 1.25)	0.148	0.98 (0.79; 1.22)	0.867^10^	1.12 (1.02; 1.23)	**0.018**	1.04 (0.93; 1.17)	0.482^10^
NPI Affective sub-syndrome	1.03 (0.96; 1.09)	0.410	1.01 (0.92; 1.11)	0.833^8^	1.09 (1.05; 1.13)	**< 0.001**	1.08 (1.03; 1.13)	**0.001** ^2^
Married	1.46 (0.64; 3.30)	0.365	0.84 (0.30; 2.39)	0.748^7^	0.69 (0.40; 1.20)	0.186	0.71 (0.38; 1.33)	0.287^7^
Entry to NH	3.57 (1.11; 11.50)	**0.033**	1.67 (0.40; 7.01)	0.488^6^	3.54 (1.64; 7.68)	**0.001**	2.52 (1.02; 6.26)	**0.047** ^4^
Assessed at baseline								
Age	0.92 (0.86; 0.98)	**0.016**	0.91 (0.84; 0.99)	**0.034** ^1^	0.95 (0.91; 0.99)	**0.021**	0.93 (0.89; 0.97)	**0.001** ^1^
Males	2.42 (1.12; 5.22)	**0.025**	2.10 (0.80; 5.53)	0.133^3^	0.68 (0.39; 1.16)	0.153	0.74 (0.40; 1.35)	0.319^9^
	Anxiolytics (N = 954)				Sedatives (N = 954)			
	Unadjusted		Adjusted		Unadjusted		Adjusted	
	OR (95% CI)	p-value	OR (95% CI)	p-value	OR (95% CI)	p-value	OR (95% CI)	p-value
Assessed at previous time-point								
CDR-SoB	1.13 (1.06; 1.21)	**< 0.001**	1.05 (0.94; 1.17)	0.377^4^	1.05 (0.99; 1.11)	0.074	0.99 (0.92; 1.08)	0.849^9^
GMHR (Good/fairly good)	0.80 (0.43; 1.51)	0.493	1.05 (0.51; 2.13)	0.901^9^	0.64 (0.41; 0.98)	**0.039**	0.70 (0.44; 1.12)	0.137^3^
PSMS	1.15 (1.06; 1.24)	**< 0.001**	1.13 (1.01; 1.26)	**0.031** ^2^	1.09 (1.03; 1.16)	**0.002**	1.08 (0.99; 1.17)	0.056^2^
NPI Agitation sub-syndrome	1.05 (0.99; 1.11)	0.089	0.98 (0.91; 1.06)	0.665^8^	1.02 (0.97; 1.07)	0.474	1.01 (0.95; 1.07)	0.813^10^
NPI Psychosis sub-syndrome	1.13 (1.01; 1.27)	**0.029**	1.06 (0.92; 1.22)	0.422^6^	1.01 (0.91; 1.12)	0.892	0.96 (0.85; 1.10)	0.576^7^
NPI Affective sub-syndrome	1.07 (1.02; 1.13)	**0.003**	1.04 (0.98; 1.10)	0.167^3^	1.03 (0.99; 1.07)	0.123	1.02 (0.98; 1.07)	0.367^5^
Married	0.91 (0.43; 1.93)	0.801	1.52 (0.65; 3.59)	0.337^5^	0.77 (0.45; 1.32)	0.336	0.77 (0.43; 1.40)	0.396^4^
Entry to NH	1.63 (0.50; 5.36)	0.420	0.67 (0.17; 2.60)	0.561^7^	1.29 (0.51; 3.23)	0.589	0.77 (0.28; 2.13)	0.608^8^
Assessed at baseline								
Age	1.02 (0.96; 1.09)	0.438	1.00 (0.94; 1.07)	0.978^10^	1.07 (1.02; 1.12)	**0.003**	1.07 (1.02; 1.12)	**0.005** ^1^
Males	0.23 (0.08; 0.69)	**0.009**	0.18 (0.05; 0.61)	**0.006** ^1^	1.00 (0.61; 1.66)	0.988	1.17 (0.67; 2.06)	0.585^6^

*CDR-SoB* Clinical Dementia Rating – Sum of Boxes

*GMHR* General Medical Health Rating, *PSMS* Physical Self-Maintenance Scale, *NPI* Neuropsychiatric Inventory, *NH* Nursing Home

### Effect of location on use of psychotropic drugs at the same time-point

**Fig. 2:**
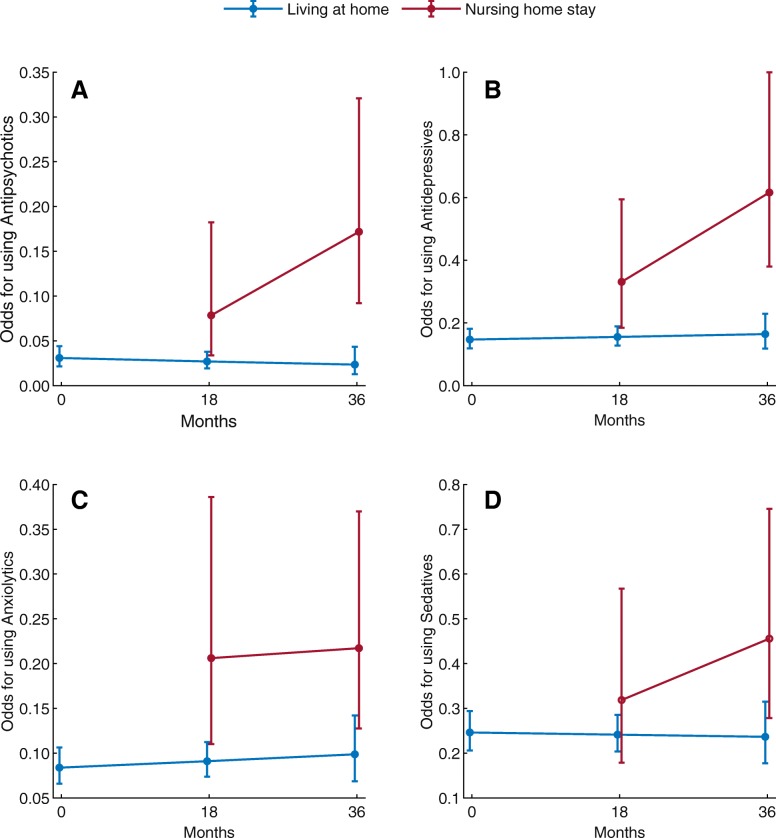
Interaction between time and location for use of Antipsychotics, Antidepressants, Anxiolytics and Sedatives

The results of effect of time and location on use of psychotropic drugs are presented in Fig. 2. In the adjusted analysis, the odds for use of antipsychotics and antidepressants were higher in those admitted to a nursing home as compared to the community-dwelling participants at A3. Please see Additional file 1: Tables S1 and S2 for further details.

More severe dementia and higher affective sub-syndrome scores, lower age and female gender were associated with higher odds for use of antidepressants (Additional file 1: Table S1). Higher affective sub-syndrome and female gender were also associated with higher odds for use of anxiolytics. Covariates associated with higher odds for use of sedatives were lower P-ADL function and increasing age, whereas good physical health (higher GMHR) decreased the odds for use of sedatives.

## Discussion

This follow-up study including 1001 community-dwelling older adults (≥ 70 years) receiving domiciliary care at baseline found that participants with dementia had higher prevalence of any psychotropic drugs than the participants without dementia. Additionally, the study revealed that those living in a nursing home at A3 had higher use of both antipsychotics and antidepressants, than the community-dwelling participants. Furthermore, persistent use of different types of psychotropic drugs was found to be high between the two consecutive assessments, both for participants with and without dementia. The persistent use diminished with longer time between assessments (Lag 2).

### Use of antipsychotics

In the present study, the prevalence of use of antipsychotics was 4% at A1 and 7% at A3. The use of antipsychotics was higher in those with dementia than in those without dementia. This is in line with previous studies including community-dwelling participants [53, 54]. Antipsychotics should never be first line treatment in people with dementia [17, 18], because of the well-known adverse effects they can cause [17–19]. However, there might be cases where it is necessary to use antipsychotics when symptoms are severe and non-pharmacological treatments are not successful [17, 18]. Unfortunately, we lack information about non-pharmacological treatments or efforts that have been tried prior to treatment with antipsychotics. Atypical antipsychotics are preferred in individuals with dementia rather than traditional antipsychotics, because of the adverse effects of traditional antipsychotics [25, 55, 56]. Even so, in the present study more than 2/3 of all participants using antipsychotics used traditional antipsychotics, and the prevalence of use of both traditional and atypical antipsychotics did not change through our study in those with dementia.

Several participants were admitted to a nursing home during the follow-up period, and when adjusting for differences in health measurements and demographics, there were elevated odds of using antipsychotics for participants that had become nursing home residents at A3. This is in line with another longitudinal study studying the transition to nursing home, and studies comparing people with dementia living in nursing homes and in the community-dwelling [36, 37, 57], However, these studies did not adjust for differences in physical health, severity of dementia and demographics between the groups. We do not have a firm explanation for the elevated use of antipsychotics in nursing home residents after adjustment for differences in health and demographics. However, it might be related to change in place of living and the stress and strain connected to transition to a nursing home [58, 59], and to the fact that nursing home residents have easier access to drug treatment than those living at home.

We found that a large percentage of participants who used antipsychotics at one time-point used the same type of drug at the following time-point. There were no differences between those with dementia and those without dementia with regard to persistent use of antipsychotics at two following time-points. This is interesting as antipsychotics, because of the mentioned side-effects, should only be used for a short period of time in people with dementia [17, 60]. Also, it is well known that antipsychotics may have a limited effect on NPS in dementia [61, 62]. The persistent use decreased when time between the two time-points increased to 36 months. This finding corresponds to the results in a longitudinal nursing home study [14]. We do not have further assessment time-points to add to our study, but it would have been interesting to explore the persistency further. We found no association between severity of psychosis sub-syndrome and use of antipsychotics at one time-point, or at two consecutive time-points. If there is no association between NPS and persistent use of antipsychotics, discontinuation should be considered in older adults with dementia. Other studies have found that antipsychotics might be discontinued without a significant increase in NPS [63]. Even so, there is need for evaluation and follow-up subsequent a discontinuation due to the risk of increased NPS after discontinuation [64].

### Use of antidepressants

In the present study, approximately 16% at A1 and 22% at A3 used antidepressants. In Norway antidepressants are commonly used in older adults, both in community dwelling and nursing home residents, and more often in females [65]. We found that the participants with dementia used antidepressants more frequently than those without dementia at all time-points and in the adjusted analysis, we found those with more severe dementia to be more likely to use antidepressants than those with less severe dementia. Furthermore, in those with more severe affective NPS the odds were higher when it came to the use of antidepressants, which was expected [66, 67]. Depressive symptoms are common in older adults with dementia [68]. However, the efficacy of antidepressant treatment of depression in people with dementia is uncertain [69]. In addition, discontinuation of antidepressants may reduce NPS and depressive symptoms in individuals with dementia [69] and are tolerated in most cases (85%) [70]. However, with discontinuation of antidepressants in participants with dementia it is essential that they are systematically and carefully monitored to identify those with increasing depressive symptoms [70].

In the adjusted analysis, nursing home residents had higher use of antidepressants at A3 compared to the community-dwelling participants at the same time-point, as reported by others [57]. Entry to nursing home may as previously mentioned increase the stress and strain on the residents and thus increase the odds for use of antidepressants, but in addition the availability to such treatment when indicated may be better than in the community-dwelling.

Not only the prevalence, but also the persistent use of antidepressants was high, both in those with and without dementia. In line with the results from a nursing home study [14], the present study found that higher severity of affective symptoms increased the odds for persistent use of antidepressants. Furthermore, we found that lower P-ADL function increased the odds for persistent use of antidepressants. We do not have a firm explanation, but it might be that those with lower P-ADL function, due to the situation of being more dependent on others, have symptoms of depression not covered by the NPI.

### Use of sedatives, anxiolytics and anti-dementia drugs

The most prevalent type of psychotropic drugs used in this sample were sedatives, with a prevalence of approximately 21–23% at the assessments. The prevalence of use of sedatives was lower in a population-based study of assumingly healthier community-dwelling older adults than the prevalence found in our study [71], but these samples are difficult to compare. For example, in the present study, use of sedatives at one time-point was associated with lower P-ADL function, poor physical health (low GMHR) and higher age, as found in other studies [72, 73].

The prevalence of anxiolytics remained quite stable at all three assessments (approximately 9–12%). The use of anxiolytics at one time-point was found to be associated with female gender and higher affective sub-syndrome of NPI at the same time-point, but persistent use of anxiolytics was not associated with affective sub-syndrome at the first time-point. Thus, this may indicate that change in affective sub-syndrome of NPI have importance for discontinuation of anxiolytics in the present study.

The prevalence of anti-dementia drugs was found to be low. However, some participants without a dementia diagnosis were using anti-dementia drugs. The explanation may be that the general practitioner usually prescribing the drugs, may prescribe the drugs because of suspicion of dementia [74]. The prevalence of anti-dementia drug use was in the descriptive analysis higher in nursing home residents, but we did not explore the use of anti-dementia drugs further, thus the findings may be due to the fact that also the prevalence of dementia was higher in nursing homes.

### Strengths and limitations

This study has significant strengths. Firstly, all nurses participating in the data collection were trained in a 2-day educational course prior to the first data collection and further one-day repetition before the second and last assessment to ensure adequate knowledge prior to the data collection. Secondly, a large sample size made it possible to adjust for many potential covariates. Lastly, it is a strength that the participants included in this study came from municipalities covering a larger part of the country and municipalities with both rural and urban areas. However, because inclusion was not based on a random selection from all parts of Norway, we cannot guarantee that the sample is representative for all older adults in Norway receiving domiciliary care.

The study has some limitations of importance. Firstly, participants were not randomly selected, and several refused to participate at baseline. In addition, our study included an expected frailer part of the community-dwelling older adults, as opposed to those without any kind of domiciliary care needs. Another inclusion criterion was that the participants had to have a next of kin that saw them at least once every week. Thus, cautions with regard generalizations of study results should be taken. Secondly, we have information about use of psychotropic drugs only from the medical record. It was not cross-checked with the Norwegian Prescription Database [65], the patient or the next of kin whether the drug still was prescribed and taken. Furthermore, we lack information about length of use and attempts of discontinuation between assessment points. Thirdly, at baseline some of the participants were already regular users of domiciliary care while others were recruited when first enrolled in the domiciliary care services. Lastly, the results of persistent use of psychotropic drugs, particularly antipsychotics, might suffer from low power, especially in the adjusted models.

### Clinical implications

It’s been known that the use of psychotropic drugs, especially antipsychotic drugs, among nursing home residents has been extensive. In Norway, as well as internationally, one has been working towards prescribing less psychotropic drugs in nursing homes. We have not had the same focus on the use of psychotropic drugs among older adults living at home, with and without dementia. Our study may contribute to broaden the focus to also include older adults, living at home with a need of domiciliary care. In such cases the GP has the responsibility to follow up the drug use, and also communication with the provider of domiciliary care so that e.g. follow-up of possible side-effects from discontinuation can be observed and registered. Less prescription of psychotropic drugs, may also introduce better treatment alternatives for NPS in older adults, e.g. milieu therapy.

## Conclusion

The prevalence and persistency of psychotropic drugs in older adults receiving domiciliary care at inclusion was high. The use of antipsychotics was higher in those with dementia than in those without dementia at all assessment time-points, and a high proportion of those using antipsychotics used traditional antipsychotics. Nursing home admission during follow-up was associated with increased odds for use of both antipsychotics and antidepressants at the last follow-up. Monitoring effects and adverse effects of psychotropic drugs is an important part of the treatment, and discontinuation of drugs should be considered regularly.

## Additional file

Additional file 1: **Tables S1,** **S2_1,** **S2**_**2,** **S2**_**3** and **S2**_**4.** **Table S1** shows effect on location on use of psychotropic drugs with covariates measured at baseline or at the same time-point. **Table S2**_**1,** **S2**_**2,** **S2**_**3** and **S2**_**4** shows interpretation of the interaction term in the model for use of Antipsychotics, Antidepressants, Anxiolytics and Sedatives, respectively. (DOCX 47 kb)

## Abbreviations

A1: Assessment at baseline
A2: Assessment 2, 18 months after baseline
A3: Assessment 3, 36 months after baseline
AIC: Akaike’s Information Criteria
ATC: Anatomical Therapeutic Chemical
CDR: Clinical Dementia Rating
CDR-SoB: Clinical Dementia Rating – Sum of Boxes
CI: Confidence Interval
GMHR: General Medical Health Rating
N: Number
NH: Nursing Home
NPI: Neuropsychiatric Inventory
NPS: Neuropsychiatric symptoms
OR: Odds Ratio
P-ADL: Personal Activities of Daily Living
PSMS: Physical Self-Maintenance Scale
SAS: Statistical Analysis Software
SD: Standard Deviation
SPSS: Statistical Package for the Social Sciences
