# Supplementary material for: Prevalence and persistent use of psychotropic drugs in older adults receiving domiciliary care at baseline
Source: BMC Geriatr. 2019 Apr 25;19:119. doi: 10.1186/s12877-019-1126-y (PMC6485106; doi:10.1186/s12877-019-1126-y)
Supplement: Supplementary file 1 — Tables S1, S2_1, S2_2, S2_3 and S2_4. Table S1 shows effect on location on use of psychotropic drugs with covariates measured at baseline or at the same time-point. Table S2_1, S2_2, S2_3 and S2_4 shows interpretation of the interaction term in the model for use of Antipsychotics, Antidepressants, Anxiolytics and Sedatives, respectively. (DOCX 47 kb) [file 12877_2019_1126_MOESM1_ESM.docx]

**Appendix Table 1**. Effect of location on use of psychotropic drugs with covariates measured at baseline or at the same time-point.

| Variable | **Antipsychotics (N=1942)** | | | | **Antidepressants (N=1942)** | | | |
| --- | --- | --- | --- | --- | --- | --- | --- | --- |
|  | Unadjusted | | Adjusted | | Unadjusted | | Adjusted | |
|  | OR (95% CI) / Coefficient (SE) | p-value | OR (95% CI) / Coefficient (SE) | p-value | OR (95% CI) / Coefficient (SE) | p-value | OR (95% CI) / Coefficient (SE) | p-value |
| *Effect of main variables*  Time  Location  Time*Location  *Assessed at previous time-point*  CDR-SoB  GMHR (Good/fairly good)  PSMS  NPI Agitation sub-syndrome  NPI Psychosis sub-syndrome  NPI Affective sub-syndrome  Married  *Assessed at baseline*  Age  Males | -0.01 (0.01)  0.15 (0.89)  0.05 (0.03)  1.11 (1.05; 1.18)  0.69 (0.42; 1.13)  1.09 (1.03; 1.15)  1.04 (1.00; 1.08)  1.07 (0.99; 1.15)  1.03 (0.99; 1.07)  1.45 (0.82; 2.56)  0.97 (0.92; 1.01)  1.76 (1.03; 3.03) | 0.462  0.869  0.083  **<0.001**  0.140  **0.003**  **0.029**  0.084  0.139  0.197  0.145  **0.040** | -0.01 (0.01)  -0.36 (0.93)  0.04 (0.03)  1.07 (0.99; 1.16)  0.86 (0.50; 1.47)  1.03 (0.96; 1.11)  1.02 (0.97; 1.06)  1.02 (0.94; 1.11)  0.99 (0.95; 1.05)  1.05 (0.56; 1.97)  0.96 (0.92; 1.01)  1.47 (0.81; 2.68) | 0.377  0.696  0.145  0.074  0.581  0.375  0.519  0.665  0.948  0.884  0.151  0.211 | 0.003 (0.006)  0.19 (0.62)  0.03 (0.02)  1.10 (1.06; 1.15)  0.76 (0.57; 1.05)  1.08 (1.04; 1.12)  1.02 (0.99; 1.05)  1.08 (1.02; 1.14)  1.07 (1.05; 1.10)  0.78 (0.53; 1.15)  0.96 (0.93; 0.99)  0.66 (0.45; 0.97) | 0.577  0.760  0.135  **<0.001**  0.096  **<0.001**  0.102  **0.007**  **<0.001**  0.204  **0.004**  **0.034** | -0.001 (0.006)  0.069 (0.65)  0.02 (0.02)  1.07 (1.01; 1.12)  0.93 (0.68; 1.29)  1.04 (0.99; 1.10)  0.97 (0.94; 1.00)  1.03 (0.96; 1.09)  1.06 (1.03; 1.09)  0.77 (0.50; 1.18)  0.94 (0.91; 0.97)  0.61 (0.40; 0.93) | 0.855  0.915  0.336  **0.013**  0.667  0.114  0.083  0.421  **<0.001**  0.229  **<0.001**  **0.021** |
| Variable | **Anxiolytics (N=1942)** | | | | **Sedatives (N=1942)** | | | |
|  | Unadjusted | | Adjusted | | Unadjusted | | Adjusted | |
|  | OR (95% CI) | p-value | OR (95% CI) | p-value | OR (95% CI) | p-value | OR (95% CI) | p-value |
| *Effect of main variables*  Time  Location  Time*Location  *Assessed at previous time-point*  CDR-SoB  GMHR (Good/fairly good)  PSMS  NPI Agitation sub-syndrome  NPI Psychosis sub-syndrome  NPI Affective sub-syndrome  Married  *Assessed at baseline*  Age  Males | 0.004 (0.006)  0.85 (0.68)  -0.002 (0.02)  1.07 (1.02; 1.11)  0.78 (0.56; 1.10)  1.06 (1.01; 1.10)  1.01 (0.98; 1.05)  1.06 (1.00; 1.13)  1.05 (1.02; 1.08)  0.86 (0.57; 1.31)  1.00 (0.97; 1.04)  0.53 (0.35; 0.82) | 0.476  0.216  0.945  **0.002**  0.154  **0.014**  0.493  **0.038**  **0.001**  0.489  0.898  **0.005** | 0.003 (0.007)  0.75 (0.71)  -0.01 (0.02)  1.04 (0.98; 1.10)  0.87 (0.61; 1.25)  1.03 (0.97; 1.09)  0.98 (0.94; 1.01)  1.03 (0.97; 1.10)  1.04 (1.01; 1.07)  1.07 (0.68; 1.68)  0.99 (0.96; 1.03)  0.49 (0.31; 0.78) | 0.634  0.288  0.661  0.208  0.449  0.348  0.210  0.324  **0.021**  0.784  0.583  **0.003** | -0.001 (0.005)  -0.10 (0.61)  0.02 (0.02)  1.03 (0.99; 1.06)  0.68 (0.52; 0.88)  1.06 (1.03; 1.10)  1.02 (0.99; 1.05)  1.02 (0.97; 1.08)  1.02 (1.00; 1.05)  0.91 (0.66; 1.26)  1.03 (1.01; 1.06)  0.94 (0.68; 1.29) | 0.815  0.870  0.306  0.114  **0.004**  **0.001**  0.097  0.484  0.052  0.560  **0.013**  0.691 | -0.002 (0.005)  -0.34 (0.63)  0.02 (0.02)  0.98 (0.94; 1.02)  0.75 (0.57; 0.98)  1.05 (1.01; 1.10)  1.02 (0.99; 1.05)  0.99 (0.93; 1.05)  1.02 (0.99; 1.04)  0.93 (0.65; 1.32)  1.03 (1.00; 1.06)  0.96 (0.68; 1.35) | 0.700  0.589  0.373  0.302  **0.036**  **0.021**  0.290  0.742  0.289  0.675  **0.024**  0.805 |

*CDR-SoB:* Clinical Dementia Rating – Sum of Boxes
*GMHR*: General Medical Health Rating
*PSMS*: Physical Self-Maintenance Scale
*NPI:* Neuropsychiatric Inventory
*Location:* The formal level of care; i.e. living at home with domiciliary care or admitted to a nursing home

**Appendix Table 2_1Antipsychotics**. Interpretation of the interaction term in the model for use of Antipsychotics

| Time | Living at home | | Living at NH | | Living at home vs Living at NH | |
| --- | --- | --- | --- | --- | --- | --- |
|  |  |  |  |  | OR (95% CI) | p-value |
| ***Antipsychotics (unadjusted)*** | | | | | | |
| A1  A2  A3 |  |  |  |  | 0.34 (0.14; 0.84)  0.14 (0.06; 0.32) | **0.019**  **<0.001** |
|  | OR (95% CI) | p-value | OR (95% CI) | p-value |  |  |
| A1 (ref.) vs A2  A1 (ref.) vs A3  A2 (ref.) vs A3 | 1.15 (0.80; 1.66)  1.32 (0.63; 2.74)  1.15 (0.80; 1.66) | 0.462  0.462  0.462 | 0.46 (0.17; 1.22) | 0.118 |  |  |
| ***Antipsychotics (adjusted)*** | | | | | | |
| A1  A2  A3 |  |  |  |  | 0.65 (0.24; 1.76)  0.29 (0.11; 0.78) | 0.392  **0.014** |
|  | OR (95% CI) | p-value | OR (95% CI) | p-value |  |  |
| A1 (ref.) vs A2  A1 (ref.) vs A3  A2 (ref.) vs A3 | 1.19 (0.81; 1.74)  1.41 (0.66; 3.02)  1.19 (0.81; 1.74) | 0.377  0.377  0.377 | 0.53 (0.19; 1.47) | 0.224 |  |  |

*NH:* Nursing home

*A1:* Assessment 1, at baseline

*A2:* Assessment 2, 18 months after baseline

*A3:* Assessment 3, 36 months after baseline

**Appendix Table 2_2 Antidepressants**. Interpretation of the interaction term in the model for use of Antidepressants

| Time | Living at home | | Living at NH | | Living at home vs Living at NH | |
| --- | --- | --- | --- | --- | --- | --- |
|  |  |  |  |  | OR (95% CI) | p-value |
| ***Antidepressants (unadjusted)*** | | | | | | |
| A1  A2  A3 |  |  |  |  | 0.47 (0.26; 0.86)  0.27 (0.15; 0.48) | **0.015**  **<0.001** |
|  | OR (95% CI) | p-value | OR (95% CI) | p-value |  |  |
| A1 (ref.) vs A2  A1 (ref.) vs A3  A2 (ref.) vs A3 | 0.95 (0.78; 1.15)  0.89 (0.60; 1.32)  0.95 (0.78; 1.15) | 0.577  0.577  0.577 | 0.54 (0.26; 1.10) | 0.089 |  |  |
| ***Antidepressants (adjusted)*** | | | | | | |
| A1  A2  A3 |  |  |  |  | 0.64 (0.33; 1.26)  0.44 (0.23; 0.86) | 0.198  **0.016** |
|  | OR (95% CI) | p-value | OR (95% CI) | p-value |  |  |
| A1 (ref.) vs A2  A1 (ref.) vs A3  A2 (ref.) vs A3 | 1.02 (0.83; 1.25)  1.04 (0.69; 1.57)  1.02 (0.83; 1.25) | 0.855  0.855  0.855 | 0.70 (0.33; 1.47) | 0.347 |  |  |

*NH:* Nursing home

*A1:* Assessment 1, at baseline

*A2:* Assessment 2, 18 months after baseline

*A3:* Assessment 3, 36 months after baseline

**Appendix Table 2_3 Anxiolytics**. Interpretation of the interaction term in the model for use of Anxiolytics

| Time | Living at home | | Living at NH | | Living at home vs Living at NH | |
| --- | --- | --- | --- | --- | --- | --- |
|  |  |  |  |  | OR (95% CI) | p-value |
| ***Anxiolytics (unadjusted)*** | | | | | | |
| A1  A2  A3 |  |  |  |  | 0.44 (0.23; 0.85)  0.45 (0.24; 0.86) | **0.015**  **0.016** |
|  | OR (95% CI) | p-value | OR (95% CI) | p-value |  |  |
| A1 (ref.) vs A2  A1 (ref.) vs A3  A2 (ref.) vs A3 | 0.92 (0.74; 1.15)  0.85 (0.54; 1.33)  0.92 (0.74; 1.15) | 0.476  0.476  0.476 | 0.95 (0.43; 2.09) | 0.897 |  |  |
| ***Anxiolytics (adjusted)*** | | | | | | |
| A1  A2  A3 |  |  |  |  | 0.57 (0.28; 1.18)  0.69 (0.33; 1.44) | 0.130  0.320 |
|  | OR (95% CI) | p-value | OR (95% CI) | p-value |  |  |
| A1 (ref.) vs A2  A1 (ref.) vs A3  A2 (ref.) vs A3 | 0.95 (0.75; 1.19)  0.89 (0.56; 1.42)  0.95 (0.75; 1.19) | 0.634  0.634  0.634 | 1.14 (0.51; 2.56) | 0.750 |  |  |

*NH:* Nursing home

*A1:* Assessment 1, at baseline

*A2:* Assessment 2, 18 months after baseline

*A3:* Assessment 3, 36 months after baseline

**Appendix Table 2_4 Sedatives**. Interpretation of the interaction term in the model for use of Sedatives

| Time | Living at home | | Living at NH | | Living at home vs Living at NH | |
| --- | --- | --- | --- | --- | --- | --- |
|  |  |  |  |  | OR (95% CI) | p-value |
| ***Sedatives (unadjusted)*** | | | | | | |
| A1  A2  A3 |  |  |  |  | 0.76 (0.42; 1.37)  0.52 (0.30; 0.89) | 0.357  **0.018** |
|  | OR (95% CI) | p-value | OR (95% CI) | p-value |  |  |
| A1 (ref.) vs A2  A1 (ref.) vs A3  A2 (ref.) vs A3 | 1.02 (0.86; 1.21)  1.04 (0.74; 1.46)  1.02 (0.86; 1.21) | 0.815  0.815  0.815 | 0.70 (0.34; 1.42) | 0.322 |  |  |
| ***Sedatives (adjusted)*** | | | | | | |
| A1  A2  A3 |  |  |  |  | 1.01 (0.53; 1.91)  0.72 (0.39; 1.33) | 0.985  0.297 |
|  | OR (95% CI) | p-value | OR (95% CI) | p-value |  |  |
| A1 (ref.) vs A2  A1 (ref.) vs A3  A2 (ref.) vs A3 | 1.04 (0.87; 1.23)  1.07 (0.76; 1.52)  1.04 (0.87; 1.23) | 0.700  0.700  0.700 | 0.74 (0.36; 1.52) | 0.415 |  |  |

*NH:* Nursing home

*A1:* Assessment 1, at baseline

*A2:* Assessment 2, 18 months after baseline

*A3:* Assessment 3, 36 months after baseline
